# Endoplasmic reticulum targeting in Ewing's sarcoma by the alkylphospholipid analog edelfosine

**DOI:** 10.18632/oncotarget.4053

**Published:** 2015-05-09

**Authors:** Ximena Bonilla, EL-Habib Dakir, Faustino Mollinedo, Consuelo Gajate

**Affiliations:** ^1^ Instituto de Biología Molecular y Celular del Cáncer, Centro de Investigación del Cáncer, Consejo Superior de Investigaciones Científicas (CSIC)-Universidad de Salamanca, Campus Miguel de Unamuno, Salamanca, Spain; ^2^ Instituto de Investigación Biomédica de Salamanca (IBSAL), Hospital Universitario de Salamanca, Salamanca, Spain; ^3^ Centre for Cancer Research and Cell Biology, Queen's University Belfast, Belfast, UK

**Keywords:** Ewing's sarcoma, endoplasmic reticulum, apoptosis, xenograft animal model, ether phospholipid, edelfosine

## Abstract

Ewing's sarcoma (ES) is the second most common bone cancer in children and young people. Edelfosine (1-*O*-octadecyl-2-*O*-methyl-*rac*-glycero-3-phosphocholine) is the prototype of a family of synthetic antitumor compounds, collectively known as alkylphospholipid analogs (APLs). We have found that APLs ranked edelfosine>perifosine>erucylphosphocholine>miltefosine for their capacity to promote apoptosis in ES cells. Edelfosine accumulated in the endoplasmic reticulum (ER) and triggered an ER stress response that eventually led to caspase-dependent apoptosis in ES cells. This apoptotic response involved mitochondrial-mediated processes, with cytochrome *c* release, caspase-9 activation and generation of reactive oxygen species. Edelfosine-induced apoptosis was also dependent on sustained c-Jun NH_2_-terminal kinase activation. Oral administration of edelfosine showed a potent *in vivo* antitumor activity in an ES xenograft animal model. Histochemical staining gave evidence for ER stress response and apoptosis in the ES tumors isolated from edelfosine-treated mice. Edelfosine showed a preferential action on ES tumor cells as compared to non-transformed osteoblasts, and appeared to be well suited for combination therapy regimens. These results demonstrate *in vitro* and *in vivo* antitumor activity of edelfosine against ES cells that is mediated by caspase activation and ER stress, and provide the proof of concept for a putative edelfosine- and ER stress-mediated approach forES treatment.

## INTRODUCTION

Ewing's sarcoma (ES) is the third most frequent primary bone cancer, and the second most common, after osteosarcoma, in children, adolescents, and young adults. ES tumors primarily affect children and teenagers, represent almost 3% of pediatric cancers with a peak incidence between the ages of 10 and 15, and are rarely seen in children younger than 5 or adults older than 30 [1-3]. ES is considered a rare disease with an estimated annual incidence of 0.6-2.9 per million population, and the natural course of the disease is rapid dissemination and death from metastases within 1-2 years from diagnosis [4].

ES is a member of Ewing's family tumors (ESFT) and it is associated in 85-90% of cases with the t(11;22)(q24:q12) chromosomal translocation that generates fusion of the 5′ segment of the EWS gene with the 3′ segment of the ETS family gene FLI-1. The resulting EWS-FLI-1 fusion protein alters transcriptional activity and modulates the expression of a number of downstream target genes relevant to apoptosis, proliferation, and neural differentiation, leading to aberrant transcription that promotes tumor initiation and propagation via prosurvival and antiapoptotic pathways [2, 5].

ES has a high metastatic proclivity and is typically refractory to conventional chemo- and radiation therapy. The prognosis of children and young adults with metastatic or recurrent disease is poor, with less than one-third of patients with metastases at diagnosis and only 10% of patients with recurrent disease being long-term survivors [6, 7]. The presence of cancer stem cells in ES might be, at least in part, responsible for refractory responses to current chemotherapies as well as for the aggressive behavior and high tendency to early metastasis and relapse of the disease [8, 9]. With modern multimodal regimens consisting of local surgery and/or radiotherapy plus intensive systemic chemotherapy, survival can be achieved for ~70% of patients with localized disease, but a relapse of ~30% leads to unfavorable prognosis [10]. However, despite advances in diagnosis and multidisciplinary treatment, the 5-year survival of ES patients with metastatic or recurrent disease remains less than 25% [10, 11], with patients with isolated lung metastases having better prognosis (30% at 5 years) than those with bone or bone marrow metastases (≤20% at 5 years) [4]. Thus, search for new chemotherapeutic agents are urgently needed to improve clinical outcome. Microarray, miRNA and functional studies suggest that ES tumors with poor prognosis show resistance to apoptosis [12-16], which might constitute one of the major mechanisms in tumor progression and chemoresistance. On these grounds, triggering of the cancer cell apoptotic machinery constitutes an appealing approach to combat ES [17].

The alkylphospholipid analogs (APLs) represent a number of structurally related compounds that show antitumor activity against several tumor cells by affecting signaling processes at the membrane level [18-21]. Among the distinct APLs, miltefosine is already in clinical use as a topical cytostatic drug for palliative treatment of cutaneous metastases from breast cancer [22, 23], and orally administered perifosine is in clinical trials for the treatment of distinct tumors [23, 24]. The ether phospholipid edelfosine (1-*O*-octadecyl-2-*O*-methoxy-*rac*-glycero-3-phosphocholine), considered as the APL prototype, is a rather selective antitumor drug [25-27] that preferentially accumulates in tumor cells [25, 26, 28, 29], and promotes apoptosis in a wide number of both hematological and solid tumor-derived cancer cells through lipid raft reorganization, leading to the activation of the death receptor Fas/CD95 independently of its natural ligand FasL/CD95L [26-28, 30-34], or by eliciting an endoplasmic reticulum (ER) stress response [34-36]. Because edelfosine is a potent inducer of apoptosis in cancer cells, we analyzed whether this proapoptotic ether lipid drug could be a promising active drug in the search of drugs targeting apoptosis in this pediatric cancer. Thus, we investigated the *in vitro* and *in vivo* action of this drug on ES cells as well as its underlying mechanism of action. In this work, we show both *in vitro* and *in vivo* evidence for the anti-ES activity of edelfosine, promoting apoptosis through the accumulation of edelfosine in the ER, leading to an ER stress response.

## RESULTS

### Edelfosine is the most active APL in promoting apoptosis in ES cells

**Figure 1:**
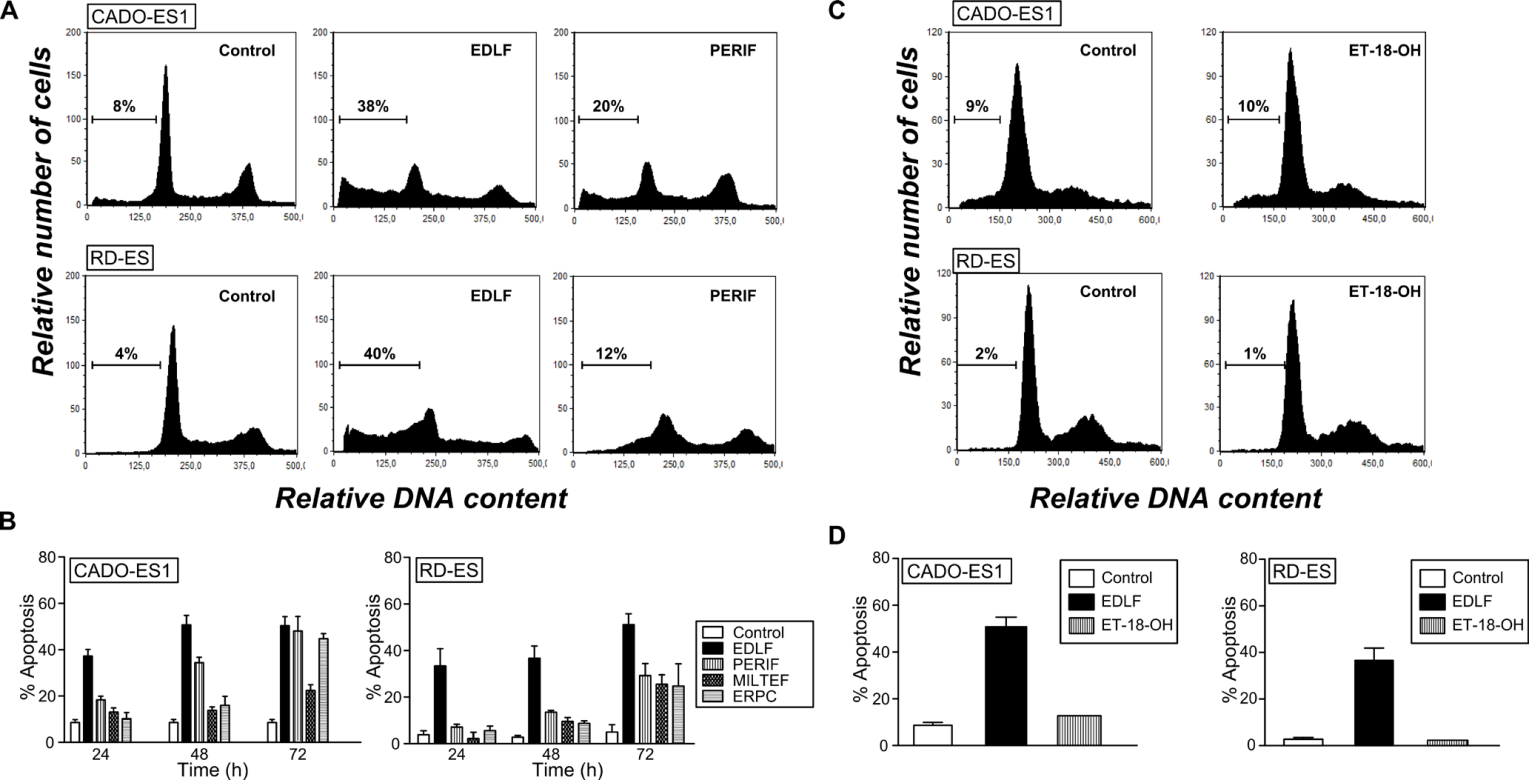
Induction of apoptosis by edelfosine and structurally related compounds in human ES cell lines **A.** Cell cycle profiles. DNA content of fixed, propidium iodide-stained cells was analyzed by flow cytometry. CADO-ES1 and RD-ES cells were incubated in the absence *(Control)* or presence of 10 μM of different APLs (edelfosine, EDLF; perifosine, PERIF) for 24 h, and then apoptosis was quantified as the percentage of cells in the sub-G_1_ region (hypodiploidy) analyzed by flow cytometry. The percentage of cells with a DNA content less than G_1_ (sub-G_1_) is indicated in each histogram. Cell cycle profiles shown, with the sub-G_1_ population indicated, are representative of three experiments performed. **B.** CADO-ES1 and RD-ES cells were incubated in the absence *(Control)* or presence of 10 μM of different APLs (edelfosine, EDLF; perifosine, PERIF; miltefosine, MILTEF; erucylphosphocholine, ERPC) for the indicated times, and the percentage of apoptosis was evaluated as the percentage of cells in the sub-G_1_ region (hypodiploidy) analyzed by flow cytometry. Data shown are means ± SD of three independent experiments. **C.** CADO-ES1 and RD-ES cells were untreated *(Control)* or treated with the inactive edelfosine analog ET-18-OH (10 μM) for 24 h, and the percentage of apoptosis was evaluated as the percentage of cells in the sub-G_1_ region (hypodiploidy) analyzed by flow cytometry. Representative cell cycle profiles of three experiments performed are shown, with the sub-G_1_ population indicated. The percentage of cells with a DNA content less than G_1_ (sub-G_1_) is indicated in each histogram. **D.** Cells were untreated *(Control)* or treated with 10 μM edelfosine (EDLF) or the inactive edelfosine analog ET-18-OH for 24 h, and then apoptosis was determined as above. Data shown are means ± SD of three independent experiments.

Previous *in vivo* and tissue distribution assays conducted in mice have shown that the pharmacologically effective concentration of edelfosine in plasma is in the 10-20 μM range [28, 29, 37]. Thus, we analyzed a time-course analysis of the ability of the most clinically relevant APLs (edelfosine, perifosine, miltefosine and erucylphosphocholine) to induce apoptosis in human CADO-ES1 and RD-ES Ewing's sarcoma cell lines when used at 10 μM. We found that edelfosine was the most active APL in eliciting an apoptotic response in both CADO-ES1 and RD-ES cells in a time-dependent manner (Figure 1A and 1B). Edelfosine was the only APL that induced a potent apoptotic response after 24 h, while the other APLs required longer incubation times (Figure 1B). APLs ranked edelfosine > perifosine > erucylphosphocholine > miltefosine for their capacity to promote apoptosis in ES cells (Figure 1B). We also found that the structurally related inactive edelfosine analog 1-*O*-octadecyl-*rac*-glycero-3-phosphocholine (ET-18-OH) [25, 38], in which the methoxy group in the *sn*-2 position of the edelfosine molecule was replaced by an OH group, was unable to trigger apoptosis in both ES cell lines (Figure 1C and 1D), indicating that the proapoptotic activity of edelfosine was highly dependent on its specific chemical structure. Because edelfosine was the most active APL against ES cells, we further analyzed the underlying mechanism of action for the anti-ES activity of edelfosine.

We also found that edelfosine induced a very weak autophagic response in ES cells, as assessed by the small rate of conversion of LC3 from the unconjugated form (LC3-I), which is in the cytosol, to the phosphatidylethanolamine-conjugated form (LC3-II) that binds to the autophagosomal membrane, as well as by the negligible effect on the BECN1 protein level (Supplementary Figure S1A). Inhibition either at the early or late stages of autophagy by using wortmannin and chloroquine, respectively, hardly affected the edelfosine-induced apoptotic response, with only a small, statistically nonsignificant increase in apoptosis in both CADO-ES1 and RD-ES cells (Supplementary Figure S1B). These data suggest that edelfosine mainly induces a potent apoptotic response in ES cells with only a minor induction of autophagy that had no consequences in the cell death response.

### ES cancer cells are more sensitive to edelfosine than non-transformed human osteoblasts

**Figure 2:**
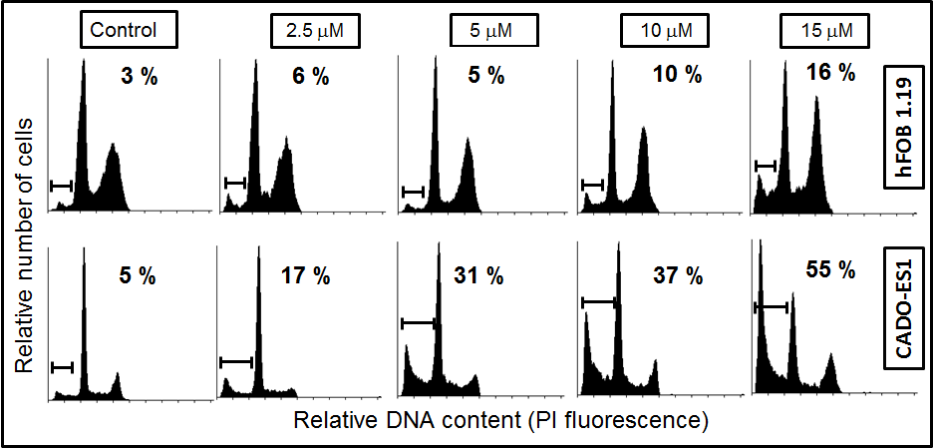
Differential induction of apoptosis in osteoblasts and ES cancer cells by edelfosine Human hFOB 1.19 osteoblasts and CADO-ES1 cancer cells were incubated with different concentrations of edelfosine for 24 h, and then apoptosis was evaluated as the percentage of cells in the sub-G_1_ region (hypodiploidy) analyzed by flow cytometry. The sub-G_1_ region is indicated in each DNA histogram, as well as the percentage of apoptosis induced after each treatment. Representative cell cycle profiles of three independent experiments are shown.

We next analyzed the proapoptotic activity of edelfosine on human hFOB 1.19 osteoblasts, which have been widely used as a model of normal osteoblasts. The hFOB 1.19 cell line was established by stable transfection of fetal bone-derived osteoblast cells with a temperature-sensitive mutant of the SV40 T antigen [39]. hFOB 1.19 cells exhibit the matrix synthetic properties of differentiated osteoblasts, and are immortalized but non-transformed human osteoblasts. This cell line is considered to be an excellent model for the study of normal osteoblast biology *in vitro* [40]. We found that higher concentrations of edelfosine were required to induce apoptosis in hFOB 1.19 cells as compared to CADO-ES1 ES cells (Figure 2), thus indicating that ES tumor cells were more sensitive to edelfosine proapoptotic action than non-transformed osteoblasts. Interestingly, CADO-ES1 cancer cells seem to be especially sensitive to edelfosine, and very low concentrations of edelfosine (2.5-5 μM) were sufficient to trigger apoptosis (Figure 2).

### Edelfosine induces a caspase-mediated apoptotic response in ES cells

**Figure 3:**
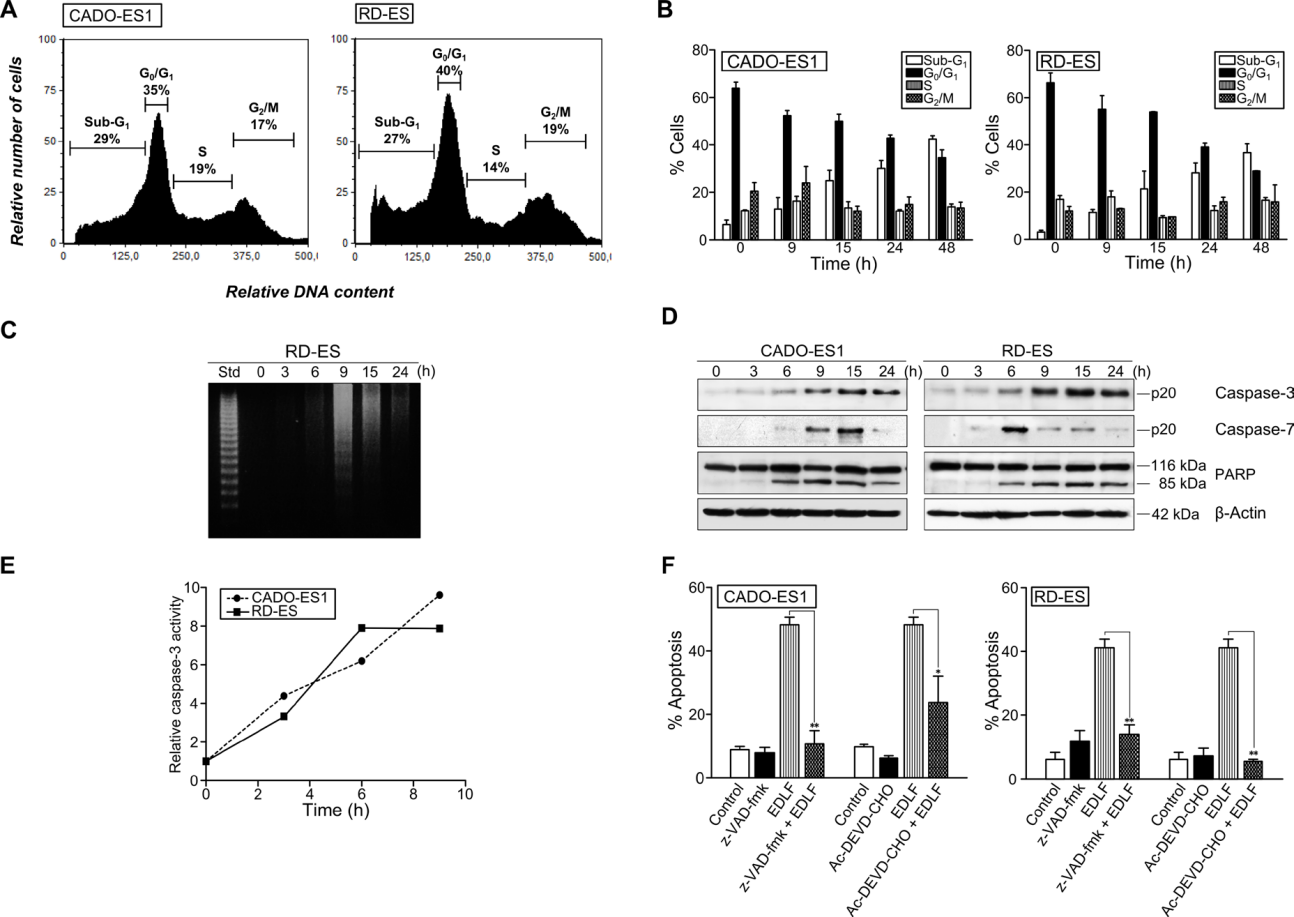
Caspase-dependent apoptosis in edelfosine-treated ES cells **A.** CADO-ES1 and RD-ES cells were incubated in the presence of 10 μM edelfosine for 24 h, and the percentages of cells at the distinct sub-G_1_ (hypodiploidy, apoptosis), G_0_/G_1_, S and G_2_/M phases were calculated by flow cytometry. Cell cycle profiles shown, with the distinct cell cycle phases indicated, are representative of three experiments performed. **B.** ES cells were treated with 10 μM edelfosine at the indicated incubation times, and the percentage of cells in each phase of cell cycle was quantitated by flow cytometry. Data shown are means ± SD of three independent experiments. **C.** RD-ES cells were untreated *(0 h)* or treated with 10 μM edelfosine at the indicated times, and then analyzed for DNA fragmentation in agarose gels. A 123-bp DNA ladder was used as standard (*Std*). Data shown are representative of three experiments performed. **D.** ES cells were incubated in the absence *(0 h)* or presence of 10 μM edelfosine for the times shown, and then cells were analyzed by immunoblotting with antibodies directed against the indicated proteins. β-Actin was used as a loading control. Data shown are representative of three experiments performed. **E.** Colorimetric measurement of caspase-3 activity following incubation of ES cells with 10 μM edelfosine for the indicated incubation times. Results show one representative experiment of three experiments performed in triplicate. **F.** ES cells, untreated *(Control)* or pre-treated with caspase inhibitors z-VAD-fmk or Ac-DEVD-CHO (50 μM) for 1 h, and then incubated without or with 10 μM edelfosine (EDLF) for 24 h, were analyzed for apoptosis by flow cytometry. Data shown are means ± SD of three independent experiments. *, *P* < 0.05; **, *P* < 0.01, Student's *t*-test.

We next characterized the apoptotic cell death induced by edelfosine in ES cells by using several *bona fide* markers of apoptosis. Cell cycle analyses by flow cytometry showed that edelfosine treatment rendered an increase in the sub-G_1_ cell population, representing apoptotic cells, whereas the cell cycle phases S and G_2_/M were not significantly affected (Figures 3A and 3B). The apoptotic response induced by edelfosine was detected after only 9-15 h incubation in both ES cell lines (Figure 3B). This apoptotic response, detected by the appearance of a sub-G_1_ population in cell cycle analysis, which is indicative of DNA degradation, was further supported by the internucleosomal DNA degradation detected after 9 h incubation with 10 μM edelfosine (Figure 3C). In addition, we found that edelfosine induced caspase-3 and -7 activation, as assessed by cleavage of procaspase-3 and -7 into their respective p20 active forms, as well as by proteolysis of the caspase-3 and -7 substrate 116 kDa-poly(ADP-ribose) polymerase (PARP) into the 85-kDa cleaved form of PARP in CADO-ES1 and RD-ES cells (Figure 3D). This caspase activation was detected at early incubation times in both ES cell lines, namely about 6 h incubation as assessed by Western blot (Figure 3D) and about 3 h incubation as estimated by colorimetric assays (Figure 3E). The pan-caspase inhibitor z-VAD-fmk as well as the caspase-3 inhibitor Ac-DEVD-CHO drastically inhibited the apoptotic death of ES cells induced by edelfosine (Figure 3F).

### Edelfosine accumulates in the ER and induces ER stress in ES cells

**Figure 4:**
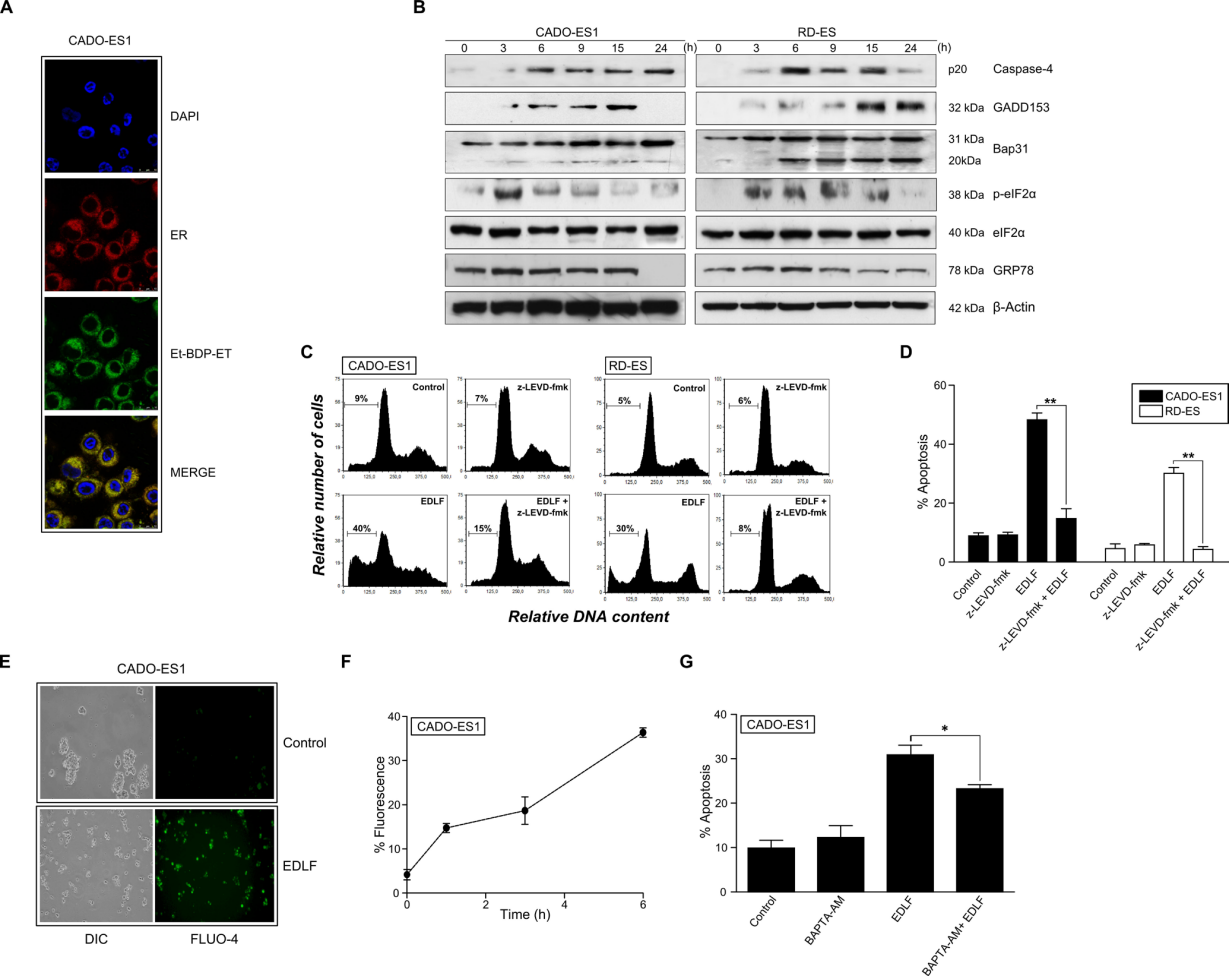
ER drug localization, ER stress and intracellular free calcium level in edelfosine-treated ES cells **A.** CADO-ES1 cells were transfected with erRFP plasmid to visualize ER (red fluorescence) and then incubated with 10 μM Et-BDP-ET (green fluorescence). Cells were also stained for nuclei with DAPI (blue fluorescence). Areas of colocalization between ER and Et-BDP-ET in the merge panels are yellow. **B.** ES cells, untreated *(0 h)* or treated with 10 μM edelfosine for the indicated times, were analyzed by Western blot using specific antibodies for the indicated proteins. β-Actin was used as a loading control. Data shown are representative of three experiments performed. **C.** Cell cycle profiles. CADO-ES1 and RD-ES cells, untreated *(Control)* or pre-treated with 5 μM z-LEVD-fmk (caspase-4 inhibitor) for 1 h, and then incubated without or with 10 μM edelfosine (EDLF) for 24 h, were analyzed for apoptosis by flow cytometry, and then the percentage of apoptosis was evaluated as the percentage of cells in the sub-G_1_ region (hypodiploidy) as in Figure 1A. Cell cycle profiles shown, with the sub-G_1_ population indicated, are representative of three experiments performed. **D.** ES cells, untreated *(Control)* or pre-treated with 5 μM z-LEVD-fmk (caspase-4 inhibitor) for 1 h, and then incubated without or with 10 μM edelfosine (EDLF) for 24 h, were analyzed for apoptosis by flow cytometry. Data shown are means ± SD of three independent experiments. **, *P* < 0.01, Student's *t*-test. **E.** CADO-ES1 cells were untreated *(Control)* or treated with 10 μM edelfosine (*EDLF*) for 3 h followed by incubation with Fluo-4 and subsequent analysis by fluorescence microscopy. DIC, differential interference contrast. **F.** CADO-ES1 cells were treated with 10 μM edelfosine for the indicated times, then incubated with Fluo-4, and fluorescence intensity was measured by flow cytometry. Data shown are means ± SD of three independent experiments. **G.** CADO-ES1 cells were preincubated without or with 10 μM BAPTA-AM for 1h, and then treated with 10μM edelfosine (*EDLF*) for 18 h and analyzed by flow cytometry to evaluate apoptosis. Data shown are means ± SD of three independent experiments. *, *P* < 0.05, Student's *t*-test.

Next, we analyzed the intracellular location of edelfosine in ES cells. To this aim, we used the fluorescent edelfosine analog 1-*O*-[11′-(6″-ethyl-1″,3″,5″,7″-tetramethyl-4″,4″-difluoro-4″-bora-3a'',4a''-diaza-s-indacen-2″-yl)undecyl]-2-O-methyl-rac-glycero-3-phosphocholine (Et-BDP-ET) that has been previously reported to be a reliable analog to visualize the subcellular localization of the drug [35, 41, 42]. Et-BDP-ET was predominately located in the ER of CADO-ES1 cells (Figure 4A), as assessed by using a version of red fluorescence protein targeted to the ER lumen (ER-targeted red fluorescence protein), which completely colocalized with the ER marker calreticulin [43]. We also found that edelfosine induced an ER stress response in both CADO-ES1 and RD-ES cell lines, as assessed by a number of ER stress markers (Figure 4B), including caspase-4 activation (cleavage of procaspase-4 into its p20 active form), CHOP/GADD153 upregulation, eIF2α phosphorylation, and cleavage of Bap31 into the 20-kDa fragment that acts as a linker of proapoptotic signals between ER and mitochondria [44]. Phosphorylation of eIF2α was detected after only 3-h incubation (Figure 4B), suggesting that edelfosine elicits rapidly an ER stress response. However, the protein level of GRP78, a major ER chaperone that promotes tumor cell survival [45], was not affected following edelfosine treatment (Figure 4B). Preincubation with the caspase-4 inhibitor z-LEVD-fmk prevented edelfosine-induced apoptosis (Figure 4C and 4D). Because ER is a key subcellular structure for Ca^2+^ storage and for the control of intracellular free Ca^2+^ levels, we examined the effect of edelfosine on intracellular Ca^2+^ levels in ES cells by using the intracellular free Ca^2+^ indicator Fluo-4 [46]. Edelfosine treatment increased the intracellular free Ca^2+^ level as detected by confocal microscopy and flow cytometry (Figure 4E and 4F). Intracellular Ca^2+^ chelation with BAPTA (1,2-bis(2-aminophenoxy)ethane-N,N,N',N'-tetraacetic acid) led to a modest, but statistically significant, reduction of the edelfosine-induced apoptotic response (Figure 4G). Taken together, these data suggest that edelfosine induces apoptosis through an ER stress response in ES cells.

### Mitochondrial involvement in edelfosine-induced apoptosis in ES cells

**Figure 5:**
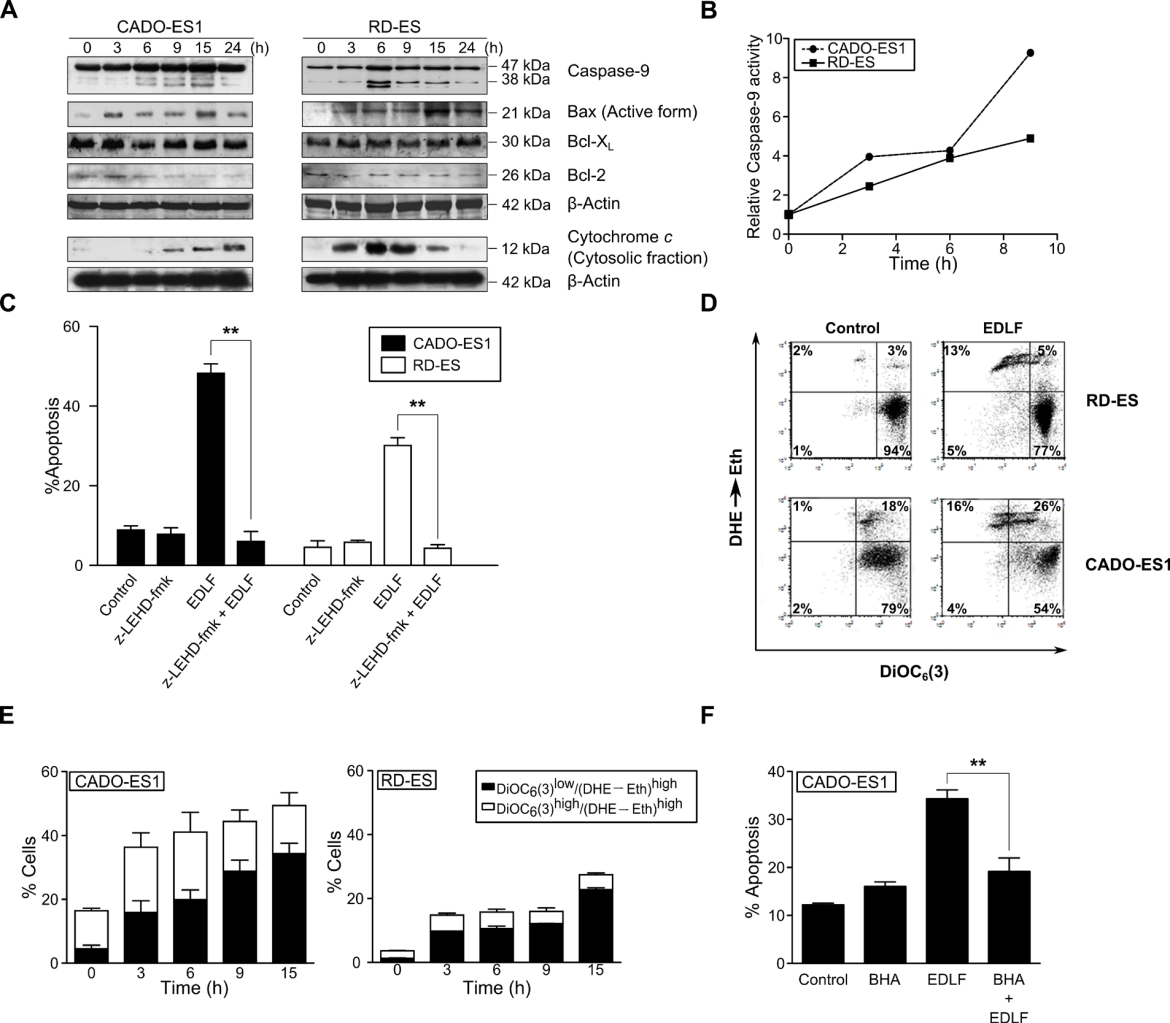
Involvement of mitochondrial-mediated signaling in edelfosine-induced apoptosis in ES cells **A.** Cells were untreated *(0 h)* or treated with 10 μM edelfosine for the times shown, and then cells were analyzed by immunoblotting with antibodies directed against the indicated proteins. β-Actin was used as a loading control. Cytochrome *c* was analyzed in the cytosolic fraction isolated as previously described [73]. Data shown are representative of three experiments performed. **B.** Colorimetric measurement of caspase-9 activity following incubation of ES cells with 10 μM edelfosine for the indicated incubation times. Results shown are representative of three experiments performed in triplicate. **C.** ES cells, untreated *(Control)* or pre-treated with 50 μM μM z-LEHD-fmk (caspase-9 inhibitor) for 1 h, and then incubated without or with 10 μM edelfosine (EDLF) for 24 h, were analyzed for apoptosis by flow cytometry. Data shown are means ± SD of three independent experiments. **, *P* < 0.01, Student's *t*-test. **D.** CADO-ES1 and RD-ES cells were incubated for 6 h in the absence *(Control)* or in the presence of 10 μM edelfosine (EDLF) and then analyzed for ΔΨ_m_ disruption [DiOC_6_(3)^low^] and ROS generation (DHE→Eth) by flow cytometry. Data shown are representative of three experiments performed. **E.** ES cells were treated with 10 μM edelfosine (EDLF) for the indicated times, and percentages of cells with disrupted ΔΨ_m_ [DioC_6_(3)^low^] and generating ROS (DHE→Eth)^high^ were measured. Data shown are means ± SD of three independent experiments. **F.** CADO-ES1 cells, untreated *(Control)* or pretreated with 50 μM BHA for 1 h, and then incubated without or with 10 μM EDLF for 24 h, were analyzed for apoptosis by flow cytometry. Data shown are means ± SD of three independent experiments. **, *P* < 0.01, Student's *t*-test.

Following ER stress response induced by edelfosine, we found Bap31 cleavage, leading to the generation of the 20-kDa fragment (Figure 4B) that acts as a signal transmitter between ER and mitochondria [44, 47]. On these grounds, we examined the putative role of mitochondria in the killing action of edelfosine on ES cells. Caspase-9 was activated by the time apoptosis was triggered, as assessed by Western blot (Figure 5A), showing the cleavage of 47 kDa-procaspase-9 into the active 35-38-kDa fragment, and by colorimetric measurements (Figure 5B). Preincubation with the caspase-9 inhibitor z-LEHD-fmk prevented edelfosine-induced apoptosis (Figure 5C). Edelfosine treatment resulted in a rapid activation of the proapoptotic protein Bax, detected by using an anti-Bax (clone 6A7) monoclonal antibody that recognized the active form of Bax (Figure 5A). However, the levels of the antiapoptotic proteins Bcl-2 and Bcl-X_L_ were hardly affected (Figure 5A). We also found a rapid release of cytochrome *c* from the mitochondria into the cytosol (Figure 5A). Edelfosine induced the generation of reactive oxygen species (ROS) as well as mitochondrial transmembrane potential (ΔΨ_m_) dissipation, as assessed by the conversion of dihydroethidium (DHE) into ethidium (Eth) (red fluorescence) and the loss of 3,3′-dihexyloxacarbocyanine iodide [DiOC_6_(3)] fluorescence (green fluorescence), respectively, in both CADO-ES1 and RD-ES cells (Figure 5D). The generation of ROS was rather prominent following incubation of ES cells with edelfosine (Figure 5D and 5E), and pretreatment of ES cells with the antioxidant butylated hydroxyanisole (BHA) inhibited edelfosine-induced apoptosis (Figure 5F), suggesting a role for ROS generation in the killing process.

### Involvement of c-Jun amino terminal kinase (JNK), but not extracellular-signal-regulated kinase (ERK), in edelfosine-induced apoptosis in ES cells

**Figure 6:**
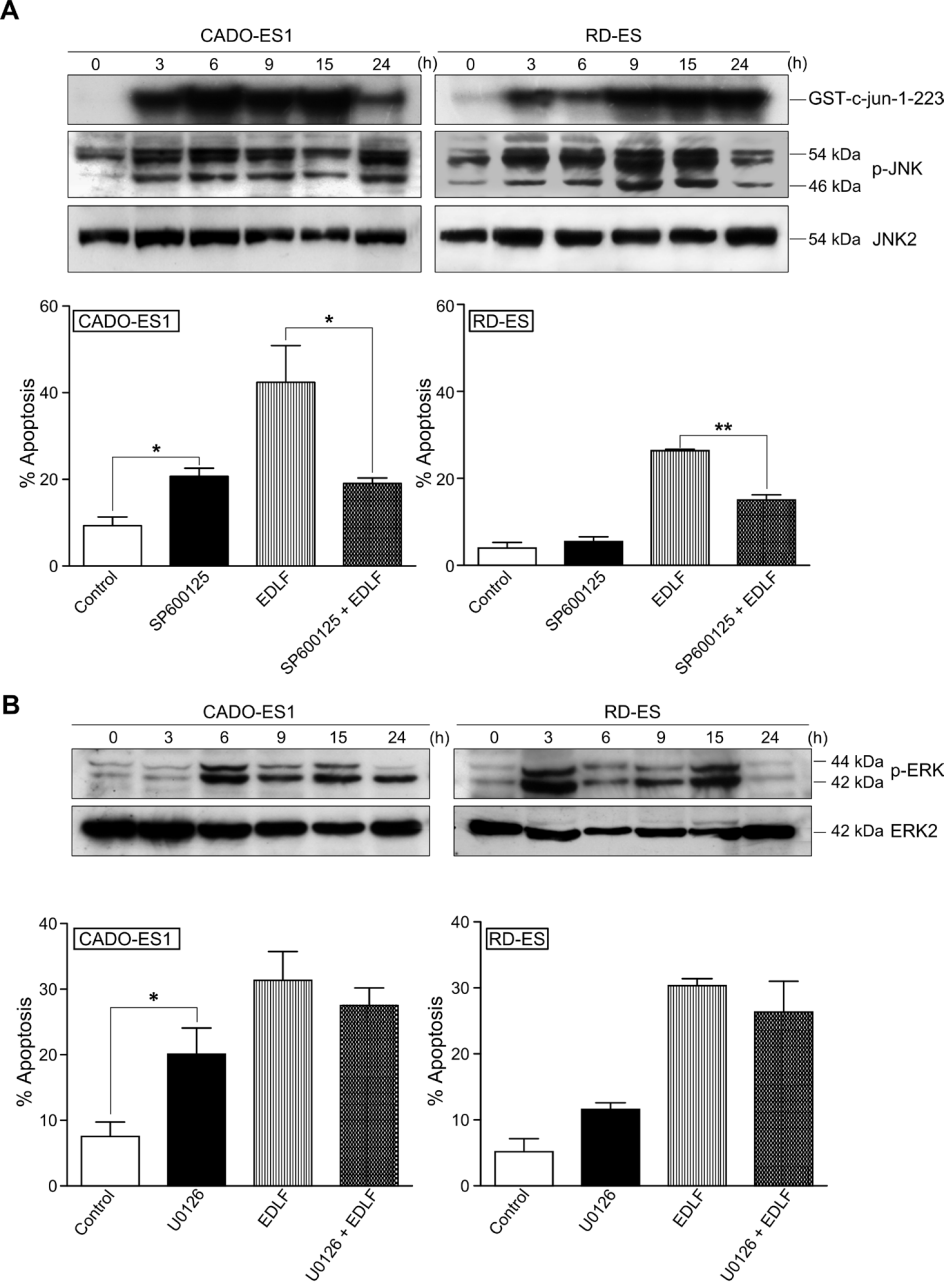
JNK and ERK activation in edelfosine-treated ES cells **A.** *(upper)* CADO-ES1 and RD-ES cells, untreated *(0 h)* or treated with 10 μM edelfosine for different times, were analyzed for JNK activation using a solid-phase JNK assay (migration position of GST-c-Jun-1-223 is indicated) and immunoblotting with antibodies directed against the indicated phosphorylated (p-JNK) (54 kDa, JNK2; 46 kDa, JNK1) and nonphosphorylated (JNK2) JNK proteins. *(lower)* ES cells, untreated *(Control)* or pretreated with 20 μM SP600125 for 1 h, and then incubated without or with 10 μM EDLF for 24 h, were analyzed for apoptosis by flow cytometry. Data shown are means ± SD *(lower)* or representative *(upper)* experiments of three performed. *, *P* < 0.05; **, *P* < 0.01, Student's *t*-test. **B.** *(upper)* CADO-ES1 and RD-ES cells, untreated *(0 h)* or treated with 10 μM edelfosine for different times, were analyzed for ERK1/2 activation by immunoblotting with antibodies directed against the indicated phosphorylated (p-ERK) (44 kDa, ERK1; 42 kD, ERK2) and nonphosphorylated (ERK2) ERK proteins. *(lower)* ES cells, untreated *(Control)* or pretreated with 10 μM U0126 for 1 h, and then incubated without or with 10 μM EDLF for 18 h, were analyzed for apoptosis by flow cytometry. Data shown are means ± SD *(lower)* or representative *(upper)* experiments of three performed. *, *P* < 0.05, Student's *t*-test.

ASK-1/JNK signaling has been implicated in ER-induced apoptosis [48]. Here, we found that edelfosine induced a potent and persistent activation of JNK in both CADO-ES1 and RD-ES cells, as assessed by a solid-phase kinase assay, using a fusion protein between GST and c-Jun (amino acids 1–223) as a substrate of JNK, as well as by Western blot for JNK phosphorylation (Figure 6A, *upper*). Using both techniques we found a very rapid activation of JNK after 3 h incubation, thus suggesting that this response was an early event following treatment of ES cells with edelfosine. Preincubation with the specific JNK inhibitor SP600125 highly reduced the edelfosine-induced apoptotic response in both CADO-ES1 and RD-ES cells (Figure 6A, *lower*). Edelfosine treatment also induced ERK phosphorylation in both ES cell lines (Figure 6B, *upper*), but preincubation with the ERK inhibitor U0126 did not significantly affect the apoptotic response induced by edelfosine (Figure 6B, *lower*), even though U0126 by itself promoted a slight increase in apoptosis, particularly in CADO-ES1 cells (Figure 6B, *lower*).

### *In vivo* antitumor effect of edelfosine in ES xenografts

**Figure 7:**
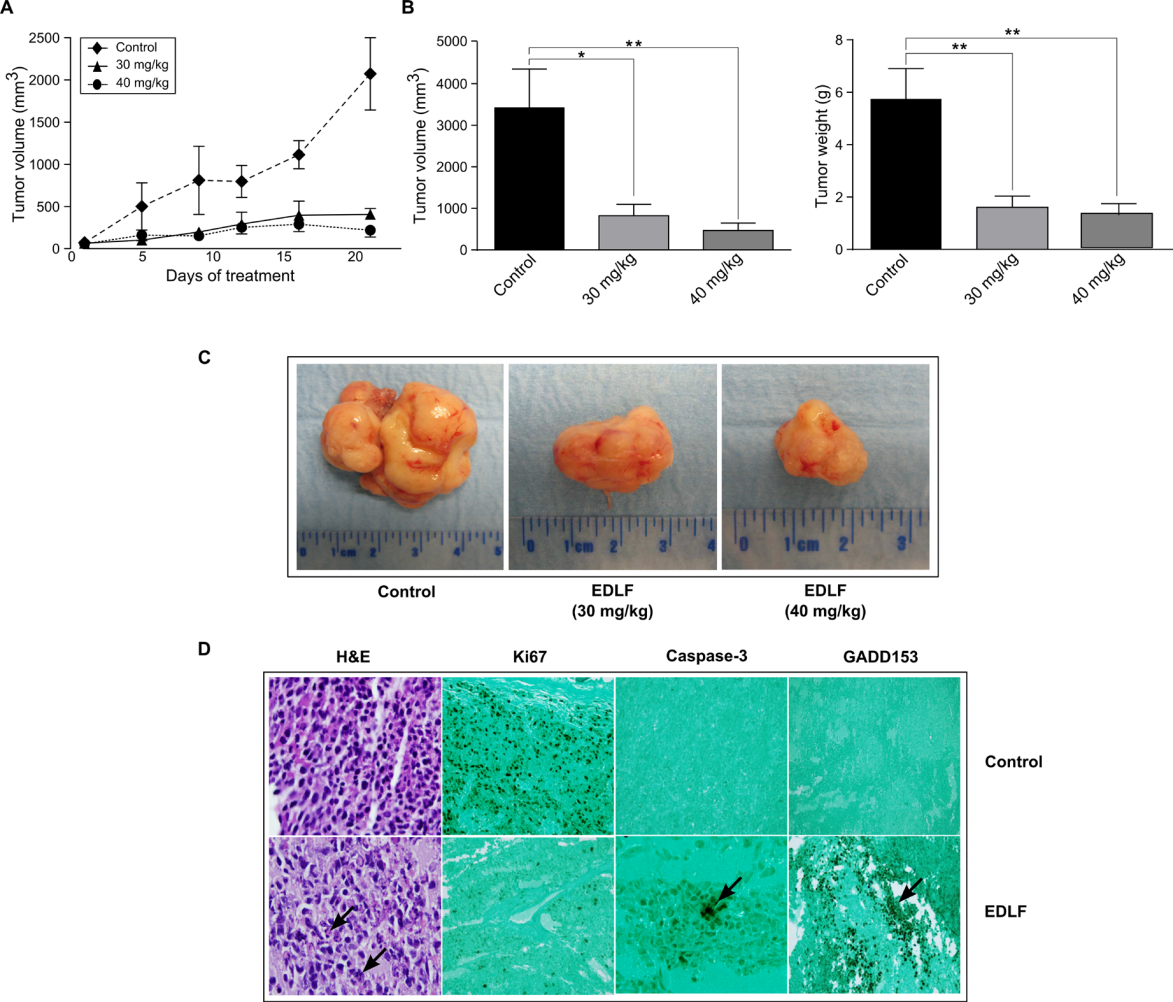
*In vivo* antitumor activity of edelfosine on human ES xenografts **A.** CB17-SCID mice were inoculated subcutaneously with CADO-ES1 cells. Oral administration of edelfosine (30 and 40 mg/kg, once-daily dosing regimen) and water vehicle *(Control)* started in parallel after the development of a palpable tumor in tumor-bearing mice (*n* = 7). Caliper measurements of each tumor were carried out at the indicated times. Data shown are means ± SD (*n* = 7). **B.** After completion of the *in vivo* assays, control drug-free mice and animals treated with 30 and 40 mg/kg edelfosine were sacrificed and tumor weight and volume were measured. Data shown are means ± SD (*n* = 7). *, *P* < 0.05; **, *P* < 0.01, Student's *t*-test. **C.** A remarkable ES tumor growth inhibition was observed after edelfosine treatment in CADO-ES1-bearing SCID mice. Representative tumors isolated from drug-free CADO-ES1-bearing mice *(Control)* and the two experimental groups of drug-treated pancreatic tumor-bearing mice (*EDLF*) are shown. **D.** Edelfosine induces apoptosis and ER stress response in ES tumor xenografts. Light microscopic and immunohistochemical examination (x200 magnification) of xenograft tumors were performed in tumor-bearing drug-free SCID mice *(Control)* and animals treated with 30 mg/kg edelfosine (EDLF). Tissue sections of tumors were stained with hematoxylin and eosin (H&E), showing the presence of irregular and medium-sized giant cells with eosinophilic cytoplasm and pyknotic nuclei *(arrow)*, indicative of cell death by apoptosis, following edelfosine treatment. Proliferative cells were observed in tumor tissue from drug-free animals following Ki67 staining, whereas the tumor sections of edelfosine-treated animals showed lack of staining. Treatment of CADO-ES1 tumor-bearing mice with edelfosine resulted in increased tumor apoptosis as detected by activated caspase-3 staining. Cleaved caspase-3-positive cells *(arrow)* were observed in edelfosine-treated mice tumors, while no staining was detected in drug-free animals. Positive staining for GADD153 is shown as dark coloring of the cell nucleus *(arrow)* in the edelfosine-treated tumor. Images shown are representative of at least four independent experiments.

We next evaluated the *in vivo* antitumor activity of orally administered edelfosine in an ES xenograft animal model. In agreement with previous studies [35], we found that daily doses of 30 mg/kg and 40 mg/kg edelfosine were well tolerated, 45 mg/kg being the maximum tolerated dose of edelfosine, in CB17-severe combined immunodeficient (SCID) mice. These mice were inoculated with 5 × 10^6^ CADO-ES1 cells. When tumors were palpable, mice were randomly assigned to three cohorts of seven mice each, receiving a daily oral administration of 30 mg/kg edelfosine, 40 mg/kg edelfosine, and vehicle (water). Serial caliper measurements were done at the indicated times to calculate the approximate tumor volume until mice were killed after a 3-week treatment (Figure 7A). Animals were killed for ethical reasons when tumor size reached ~10% of body weight, and this size was approximately reached by 3 weeks time from the start of treatment in the ES tumor-bearing mice (Figure 7A). A comparison of tumors isolated from untreated control and drug-treated CADO-ES1-bearing mice, at the end of the treatment, rendered a remarkable anti-ES activity of edelfosine (Figure 7B and 7C), with a statistically significant reduction in both tumor weight and volume (Figure 7B). The 30 mg/kg edelfosine treatment rendered a reduction of 73% and 75% in tumor weight and volume respectively, whereas the treatment of 40 mg/kg edelfosine led to a reduction of 77% and 86% in tumor weight and volume respectively (Figure 7B). No morphological alterations were observed in the organs of drug-treated mice as compared to drug-free control animals, indicating no apparent toxicity. Likewise, no significant differences in body weight were found between drug-treated and untreated control animals (less than 3% of body weight loss in drug-treated *vs.* control cohorts).

### *In vivo* identification of apoptosis and ER stress in ES tumors following edelfosine oral treatment

Histological patterns of tumors isolated from control drug-free tumor-bearing animals revealed a relative uniformity in cell size and morphology, including the nuclear morphology. In contrast, examination of hematoxylin and eosin-stained sections of tumors from mice orally treated with edelfosine showed the presence of irregular, large and medium-sized giant cells with eosinophilic cytoplasm and pyknotic nuclei (Figure 7D). The nuclei in dying cells displayed nuclear fragmentation characteristic of cell death by apoptosis (Figure 7D). A strong cell proliferation activity, as assessed by Ki67 staining, was determined in the tumors isolated from the control drug-free group (Figure 7D), but no cell proliferation was detected in the tumor xenografts from the edelfosine-treated group (Figure 7D). The anti-activated caspase-3-specific antibody selectively labeled the cytoplasm of apoptotic cells in tumors from drug-treated mice, while no staining was observed in the control drug-free group (Figure 7D). Upregulation of the ER stress-associated marker CHOP/GADD153, indicating an ER stress response, was visualized in the ES tumors isolated from edelfosine-treated mice, but not from control drug-free mice (Figure 7D). These results indicate that edelfosine treatment induces ER stress and apoptosis in the ES tumors *in vivo*.

### Combination of edelfosine with drugs in clinical use for ES potentiates apoptosis

**Figure 8:**
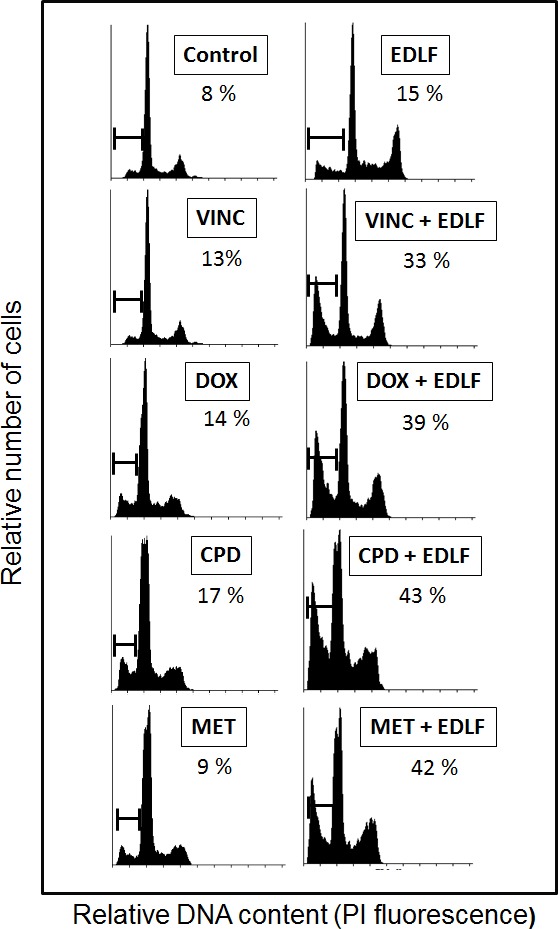
The combination of edelfosine with different antitumor agents potentiates apoptosis in ES cells CADO-ES1 cells were incubated for 24 h with 2.5 μM edelfosine (EDLF), 1 nM vincristine (VINC), 1 nM doxorubicin (DOX), 500 μM cyclophosphamide (CPD), 100 μM metformin (MET) or the combination of edelfosine with the above agents. Untreated control cells were run in parallel. Apoptosis was determined by flow cytometry as the percentage of hypodiploid (sub-G_1_) cells following cell cycle analysis. The percentage of cells with a DNA content less than G_1_ (sub-G_1_) is indicated in each histogram. Cell cycle profiles shown, with the sub-G_1_ population indicated, are representative of three experiments performed.

We next analyzed the putative potentiation of apoptosis as a result of combining edelfosine with additional drugs used in the clinics for ES treatment and acting through different mechanisms of action. Current standard chemotherapy includes vincristine, doxorubicin, and cyclophosphamide, also known as VDC, alternating with ifosfamide and etoposide [49, 50]. As shown in Figure 8, we found that the combination of edelfosine with vincristine, doxorubicin or cyclophosphamide potentiated the proaptototic effect of edelfosine in CADO-ES1 cancer cells. Vincristine as well as other microtubule-damaging agents suppress microtubule dynamics, leading to disruption of the mitotic spindle in dividing cells, cell cycle arrest and apoptosis [51, 52], whereas doxorubicin and cyclophosphamide act through DNA interaction [53, 54]. Because edelfosine does not target DNA, but acts at the cell membrane level [18, 33, 34], this drug is a good candidate for the combination with the above antitumor drugs in order to improve therapeutic regimens for the treatment for this pediatric cancer. Interestingly, we have also found that the combination of metformin and edelfosine also potentiates apoptosis in CADO-ES1 cells (Figure 8). Metformin is the most commonly prescribed drug for type 2 diabetes mellitus, but it is now emerging as an attractive candidate for cancer prevention and treatment [55].

## DISCUSSION

The results reported here show for the first time the antitumor activity of the ether phospholipid edelfosine against ES cells, and point out the involvement of ER as a target in ES therapy. Edelfosine induces caspase-dependent apoptotic cell demise in ES cells that is mainly mediated by targeting ER and involves ER stress and mitochondrial-mediated processes. Our data indicate that edelfosine is the most potent APL in eliciting an apoptotic response in ES cells. Among the most clinically relevant APLs, we found that these compounds ranked edelfosine > perifosine > erucylphosphocholine > miltefosine for their capacity to promote apoptosis in ES cells. Edelfosine induced a remarkable apoptotic activity in ES cells when used at the low micromolar range (5-10 μM), which fits well with the steady-state plasma levels of edelfosine (10-20 μM) found in a number of animal model *in vivo* assays [28, 29, 37]. In addition, ES cancer cells seem to be rather sensitive to edelfosine, as apoptosis was triggered upon incubation with very low concentrations of edelfosine (2.5-5 μM). The fact that ES tumor cells were more sensitive to edelfosine than the non-transformed hFOB 1.19 osteoblast cell line is in agreement with previous reports showing a rather selective proapoptotic action of edelfosine on tumor cells [25-27].

**Figure 9:**
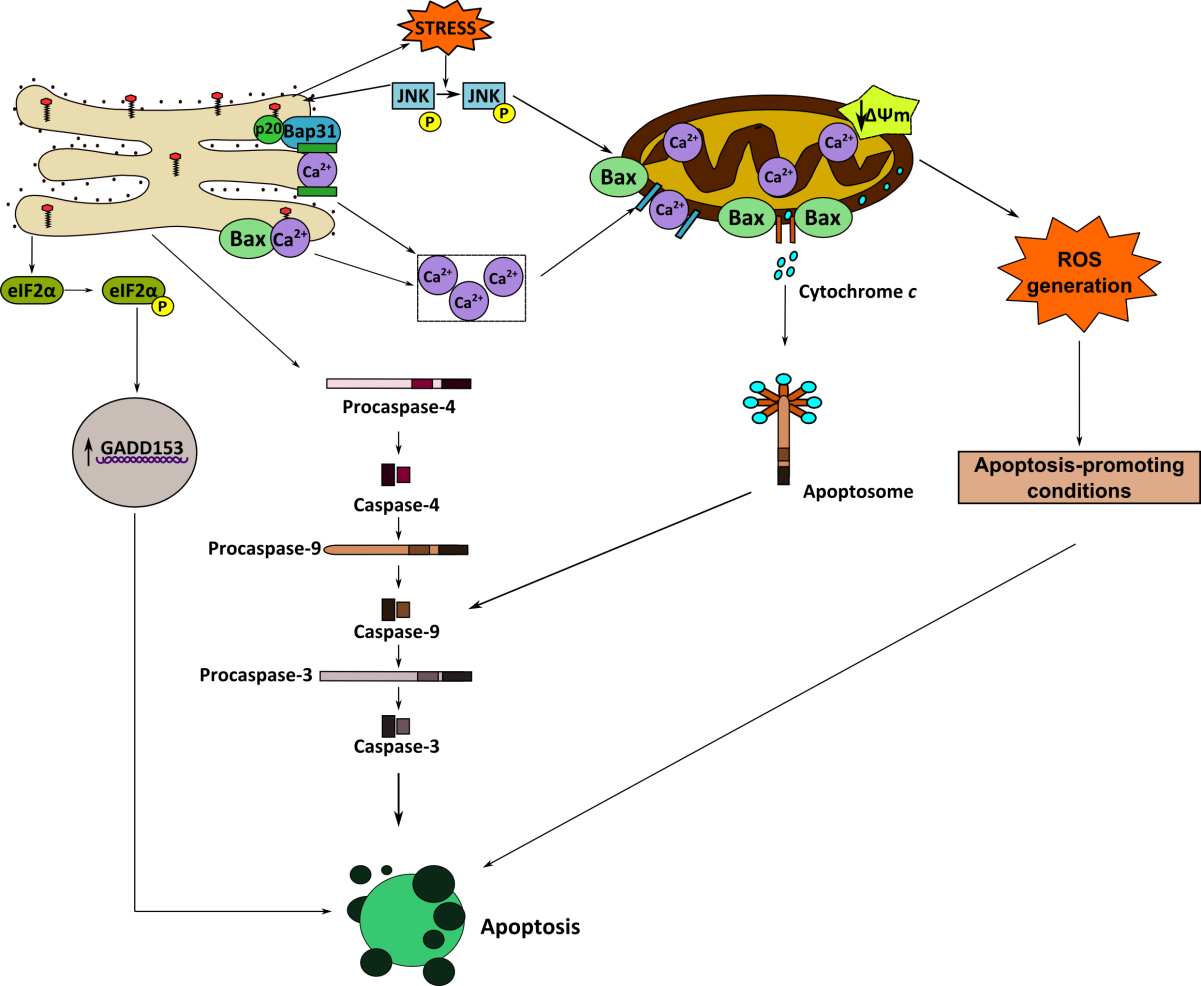
Schematic model of ER involvement in edelfosine-induced apoptosis in ES cancer cells This is a schematic diagram designed to portray one currently plausible mechanism of how edelfosine induces apoptosis via ER stress in ES tumor cells. Edelfosine accumulates in the ER, inducing an ER stress response that leads to cell death. See text for details.

Figure 9 depicts a schematic model of a putative mechanism of action for edelfosine-induced apoptosis in ES cells. Edelfosine accumulates in the ER where it triggers an ER stress response, leading to caspase-4 activation, CHOP/GADD153 upregulation, eIF2α phosphorylation, increase in intracellular free Ca^2+^ level, and cleavage of Bap31 into the 20-kDa fragment, which directs proapoptotic signals between ER and mitochondria [44]. Mitochondria seem to be critical in the apoptotic outcome in edelfosine-treated ES cells, leading to Bax and caspase-9 activation, ΔΨ_m_ dissipation, release of cytochrome *c* to the cytosol, and ROS generation. Caspase-9 could be activated by either ER-related caspase-4 activation [56] or apoptosome formation through the binding of cytochrome *c* to apoptosis protease-activating factor-1 [57]. Inhibition of caspase-4, caspase-9 and ROS generation blocked edelfosine-induced apoptosis, highlighting the essential role of ER and mitochondria in edelfosine-induced apoptosis in ES cells. The generation of ROS is suggested to lead to the acquisition of apoptosis-promoting conditions, thus facilitating the triggering of an apoptotic response. Increased intracellular free Ca^2+^ level seems to play a role, but likely not an essential one, in edelfosine-induced cell demise, as Ca^2+^ chelation inhibits, but does not totally block, apoptosis. JNK was persistently activated during incubation of ES cells with edelfosine, and JNK inhibition prevented edelfosine-induced apoptosis, suggesting a major role for JNK in the proapoptotic action of edelfosine on ES cells, in a similar way as previously found for the proapoptotic action of edelfosine in leukemic [38] and pancreatic [35] cancer cells. Thus, JNK seems to be a critical signaling pathway in the proapoptotic action of edelfosine in cancer cells.

The results reported here represent the first study on the action of APLs in ES and demonstrate the potent proapoptotic *in vitro* and *in vivo* anti-ES activity of edelfosine. In addition, the upregulation of CHOP/GADD153 in the xenograft tumors that undergo apoptosis following edelfosine oral treatment of ES tumor-bearing mice further supports the *in vivo* ER stress response induced by edelfosine in this tumor. Perifosine has been recently found to induce apoptosis in human osteosarcoma cells [58]. A phase II study of daily oral perifosine in patients with advanced soft tissue sarcoma showed prolonged responses in heavily pretreated patients, even though the primary objective of ≥40% 6-month progression-free survival rate was not achieved, with grade 1 to 2 gastrointestinal toxicity or fatigue being the most common (> 50% of patients) toxicities observed [59]. In contrast, another phase II study involving adult patients with locally advanced or metastatic inoperable soft tissue sarcoma did not show evidence of activity [60]. Our data suggest that edelfosine shows a higher anti-ES activity than perifosine, and previous animal model studies have shown the lack of significant toxicity following orally administered edelfosine [28, 29, 61], including lack of cardiotoxicity, hepatotoxicity or renal toxicity [61]. Our present study on the *in vitro* and *in vivo* actions of edelfosine on ES, together with the effective *in vivo* antitumor action of this APL on several additional tumors, such as pancreatic cancer [35], multiple myeloma [28], mantle lymphoma [29] and chronic lymphoblastic lymphoma [29], warrants further studies on the putative use of edelfosine as a promising antitumor drug lacking significant toxicity.

Interestingly, the herein reported potentiation of proapoptotic activity following combination of edelfosine with several anticancer drugs currently used for ES treatment suggests that edelfosine could be a promising candidate for the use of combination therapy in this disease. The results reported here suggest that the combined use of edelfosine, acting at the membrane level, with drugs targeting microtubules (vincristine) or DNA (doxorubicin, cyclophosphamide) might improve the current therapeutic regimens for ES. Furthermore, we also found here that the combined treatment of metformin and edelfosine also potentiated apoptosis in ES cells. Metformin, a well-known insulin-sensitizer commonly used for type 2 diabetes therapy that is gaining attention in cancer research [62-64], has recently been found to inhibit growth and sensitize osteosarcoma cell lines to cisplatin [65]. In addition, metformin has been recently found to potentiate the efficacy of antitumor drugs against different tumor cells [66-70]. Our results have shown that the combined treatment of edelfosine and the above drugs noticeably potentiated the apoptosis outcome elicited by each individual drug in ES cells, thus suggesting that edelfosine, as well as its underlying endoplasmic reticulum-mediated mechanism of action, might be of interest for combination therapy regimens in the treatment of this pediatric cancer.

Despite multimodal therapy, consisting of high-dose chemotherapy, surgery and radiotherapy, survival of patients with metastatic or recurrent ES has not improved significantly during the past decades. The results reported here provide the proof of concept for the anti-ES efficacy of edelfosine, and suggest that edelfosine oral treatment can become a promising new drug for ES therapy. On these grounds, further studies focused on effectively translating the herein reported therapeutic benefits in a putative clinical setting are warranted. In addition, the fact that edelfosine targets ER in ES cells (this report) as well as in pancreatic cancer cells [35], leading to ER stress-mediated apoptosis, highlights that ER can be a promising and valuable target for the design of therapeutic strategies and drugs aimed to improve cancer treatment, and particularly ES prognosis.

## MATERIALS AND METHODS

### Drugs and reagents

Edelfosine was obtained from R. Berchtold (Biochemisches Labor, Bern, Switzerland). A stock solution was prepared at 2 mM in RPMI-1640 culture medium, containing 10% (v/v) fetal bovine serum (FBS), by heating at 50°C for 30 min, as previously described [25]. Perifosine (octadecyl-(1,1-dimethyl-piperidinio-4-yl)-phosphate) and erucylphosphocholine ((13Z)-docos-13-en-1-yl 2-(trimethylammonio)ethyl phosphate) were from Zentaris. Miltefosine (hexadecylphosphocholine) was from Calbiochem. Stock sterile solutions of the distinct APL analogs (2 mM) were prepared in RPMI-1640 culture medium (Invitrogen), supplemented with 10% (v/v) heat-inactivated fetal bovine serum (FBS), 2 mM L-glutamine, 100 U/ml penicillin, and 100 μg/ml streptomycin (GIBCO-BRL), as above. Caspase-4 inhibitor z-LEVD-fmk and the pan-caspase inhibitor z-VAD-fmk were from Alexis Biochemicals. Caspase-3 inhibitor Ac-DEVD-CHO, caspase-9 inhibitor z-LEHD-fmk, and the JNK inhibitor SP600125 were from Calbiochem. Acrylamide, bisacrylamide, ammonium persulfate, and N,N,N'N'-tetramethylethylenediamine were from Bio-Rad. All other chemicals were from Merck or Sigma.

### Cells and culture conditions

The human ES cell lines CADO-ES1 and RD-ES were from the Deutsche Sammlung von Mikroorganismen und Zellkulturen GmbH-DSMZ (German Collection of Microorganisms and Cell Cultures), and were grown in RPMI-1640 supplemented with 10% (v/v) heat-inactivated FBS, 2 mM L-glutamine, 100 U/ml penicillin, and 100 μg/ml streptomycin at 37ºC in humidified 95% air and 5% CO_2_. APLs were added to the cell culture at the concentrations and for the times indicated in the respective figures. To analyze the effect of the distinct caspase and kinase inhibitors used in this study, 2.5 × 10^5^/ml cells were pretreated with each inhibitor for 1 h before edelfosine treatment, and then samples were analyzed by flow cytometry to determine apoptosis through cell cycle analyses.

### Measurement of caspase activity by colorimetric methods

Caspase-3 and caspase-9 activities were quantified by spectrophotometric detection of chromophore (pNA, p-nitroanilide) following its cleavage from the labeled substrates Ac-DEVD-pNA and Ac-LEHD-pNA (Calbiochem), respectively. ES cell lines were treated with 10 μM edelfosine during different time periods. Then cells were lysed with extract buffer (25 mM HEPES, pH 7.7, 0.3 M NaCl, 1.5 mM MgCl_2_, 0.2 mM EDTA, 0.1% Triton X-100, 20 mM β-glycerophosphate, 0.1 mM Na_3_VO_4_), containing protease inhibitors (1 mM PMSF, 20 μg/ml aprotinin and 20 μg/ml leupeptin) and supplemented with 2 mM DTT. For the quantification of caspase activity, 25 and 50 μg protein were used for evaluation of caspase-3 and caspase 9 activities respectively in 96-well plates. Next, extracts were incubated with 100 μM of substrate in the reaction buffer (25 mM HEPES, pH 7.4, 10% sucrose, 10 mM DTT) for 1 h at 37°C. All reactions were performed in triplicate. The samples were read by an ELISA spectrophotometer (BioRad) at 405 nm. The results are expressed as relative caspase activity, which is the ratio between the caspase activity of the sample and that measured in control (without treatment).

### Apoptosis assays

Quantification of apoptotic cells was determined by flow cytometry as the percentage of cells in the sub-G_1_ region (hypodiploidy) in cell cycle analysis as previously described [26]. Cell cycle profiles were generated using manually drawn gates with Cyflogic software. Apoptosis was also assessed by isolation of fragmented DNA and subsequent analysis by electrophoresis on 1% agarose gels as previously described [25, 71]. A 123-bp DNA ladder (Life Technologies) was used as standard. DNA was visualized after electrophoresis by ethidium bromide staining.

### Solid phase JNK assay

A fusion protein between glutathione S-transferase (GST) and c-Jun (amino acids 1-223) was used as a substrate for JNK as previously described [38, 72]. The ^32^P-phosphorylated proteins were resolved in SDS-10% polyacrilamide gels followed by autoradiography.

### Western blot analysis

5 × 10^6^ cells were lysed with 60 μl of extract buffer (25 mM HEPES, pH 7.7, 0.3 M NaCl, 1.5 mM MgCl_2_, 0.2 mM EDTA, 0.1% Triton X-100, 20 mM β-glycerophosphate, 0.1 mM Na_3_VO_4_) supplemented with protease inhibitors (1 mM phenylmethylsulfonyl fluoride, 20 μg/ml aprotinin, 20 μg/ml leupeptin). Forty five micrograms of proteins were run in SDS polyacrylamide gels, transferred to nitrocellulose filters, blocked with 5% (w/v) power defatted milk in TBST (50 mM Tris-HCl (pH 8.0), 150 mM NaCl, 0.1%Tween 20) for 90 min at room temperature, and incubated for 1 h at room temperature or overnight at 4ºC with the following specific antibodies: anti-active caspase-3 rabbit monoclonal antibody (1:1000, BD Pharmingen), anti-poly(ADP-ribose) polimerase mouse monoclonal antibody (1:1000, BD Pharmingen), anti-caspase-7 mouse monoclonal antibody (1:1000, BD Pharmingen), anti-caspase-9 rabbit polyclonal antibody (1:1000, Oncogene), anti-caspase-4 goat polyclonal antibody (1:250, Santa Cruz Biotechnology), anti-GADD153 rabbit monoclonal antibody (1:250, Santa Cruz Biotechnology), anti-Bap31 (C-15) goat polyclonal antibody (1:500, Santa Cruz Biotechnology), anti-eIF2α rabbit polyclonal antibody (1:1000, Cell Signaling Technology), anti-phospho-eIF2α (Ser51) rabbit monoclonal antibody (1:1000, Cell Signaling Technology), anti-GRP78 rabbit monoclonal antibody (1:1000, Cell Signaling Technology), anti-Bcl-2 mouse monoclonal antibody (1:500, BD Pharmingen), anti-Bcl-X_L_ monoclonal antibody (1:1000, Cell Signaling Technology), anti-p-JNK (Thr183/Tyr185) (98F2) and anti-JNK2 rabbit monoclonal antibodies (1:1000, Cell Signaling Technology), anti-p- ERK1/2 rabbit polyclonal antibody (1:1000, Cell Signaling Technology), anti-ERK2 mouse monoclonal antibody (1:1000, Santa Cruz Biotechnology), anti-LC3 (microtubule-associated protein 1 light chain 3) rabbit polyclonal antibody (1:1000, Cell Signaling Technology), anti-BECN1 (Beclin-1; Bcl-2 interacting myosin/moesin-like coiled-coil protein 1) rabbit polyclonal antibody (1:1000, Cell Signaling Technology). Signals were developed using an enhanced chemoluminescence detection kit (*GE* Healthcare).

### Mitochondrial cytochrome *c* release

Release of cytochrome *c* from mitochondria to cytosol was analyzed by Western blot of the cytosolic fractions as previously described [73], using an anti-cytochrome *c* (7H8.2C12) mouse monoclonal antibody antibody (1:500, BD Pharmingen).

### ER localization of edelfosine

The subcellular localization of edelfosine in the ER of ES cells was carried out as previously described [35]. 5 × 10^5^ cells were transfected with a plasmid encoding endoplasmic reticulum-targeted red fluorescence protein (erRFP) [43], kindly provided by F. X. Pimentel-Muinos (Instituto de Biología Molecular y Celular del Cáncer, Centro de Investigación del Cáncer, Salamanca, Spain), using Lipofectamine 2000 transfection reagent (Invitrogen), and then incubated for 1 h with 10 μM of the fluorescent edelfosine analog Et-BDP-ET [42], kindly provided by F. Amat-Guerri and A. U. Acuña (CSIC, Madrid, Spain). Colocalization of the distinct fluorochromes was analyzed using a LSM 510 laser scan confocal microscope (Carl Zeiss).

### Analysis of ΔΨ_m_ and ROS generation by flow cytometry

To evaluate ΔΨ_m_ and ROS generation, 2.5 × 10^5^ cells were washed once with PBS and then were incubated in 500 μl PBS with 20 nM DiOC_6_(3) (green fluorescence) (Molecular Probes) and 2 μM DHE (red fluorescence after oxidation) (Sigma) for 20 min at 37ºC, followed by analysis on a FACSCalibur flow cytometer (Becton Dickinson) as previously described [74].

### Measurement of intracellular calcium

Cells were incubated with 2 μM Fluo-4 AM dye (Molecular Probes) for 30 min at 37ºC, and then analyzed by fluorescence microscopy or flow cytometry.

### Xenograft mouse models

Animal procedures in this study complied with the Spanish (Real Decreto RD1201/05) and the European Union (European Directive 2010/63/EU) guidelines on animal experimentation for the protection and humane use of laboratory animals, and were conducted at the accredited Animal Experimentation Facility (Servicio de Experimentación Animal) of the University of Salamanca. Procedures were approved by the Ethics Committee of the University of Salamanca. Female CB17-SCID mice (8-week old) (Charles River Laboratories), kept and handled according to institutional guidelines, complying with Spanish legislation under 12/12 h light/dark cycle at a temperature of 22°C, received a standard diet and acidified water *ad libitum*. CADO-ES1 cells (5 × 10^6^) were injected subcutaneously in 100 μl phosphate-buffered saline together with 100 μl Matrigel basement membrane matrix (Becton Dickinson) into the right flank of each mouse. When tumors were palpable, approximately 2 weeks after tumor cell implantation, mice were randomly assigned to cohorts of seven mice each, receiving a daily oral administration of edelfosine (30 or 40 mg/kg of body weight) or an equal volume of vehicle (water). The shortest and longest diameter of the tumor were measured with calipers at the indicated time intervals, and tumor volume (mm^3^) was calculated using the following standard formula: (the shortest diameter)^2^ × (the longest diameter) × 0.5. Animal body weight and any sign of morbidity were monitored. Drug treatment lasted 3 weeks. Animals were killed 24 h after the last drug administration according to institutional guidelines, and then tumors were carefully removed, weighed and analyzed. A necropsy analysis involving tumors and distinct organs was carried out.

### Histochemical analysis

Tumor tissue samples were fixed in 4% buffered paraformaldehyde and embedded in paraffin. Tissue sections (5 μm) were deparaffinized and hydrated in graded ethanol and distilled water. Endogenous peroxidase activity was blocked using methanol and 30% H_2_O_2_ for 30 min. Sections were counterstained with hematoxylin and eosin (H&E), and then incubated overnight at 4ºC with different antibodies: rabbit anti-human Ki67 monoclonal antibody (clone SP6) (1:1000 dilution, Abcam), anti-active human caspase-3 (1:100 dilution) rabbit polyclonal antibody that specifically recognized the active ~20-kDa subunit (Cell Signaling Technology), or with F-168 anti-human CHOP/GADD153 (1:50 dilution) rabbit polyclonal antibody (Santa Cruz Biotecnology). After washing with PBS, sections were incubated with biotinylated anti-rabbit IgG antibody (BD Pharmingen) for 60 min at room temperature and washed with PBS. Then, sections were incubated with streptavidin horseradish-peroxidase (Vector Laboratories) for 60 min in a moist chamber, washed with PBS, and sites of peroxidase activity were visualized using 3,3′-diaminobenzidine tetrahydrochloride solution (DAB). Sections were subsequently counterstained with Mayer's hematoxylin or Light Green. Staining was analyzed with a Nikon Eclipse 400^®^ microscope and Metamorph^®^ software (Molecular Devices Corporation).

### Statistical analysis

Results are shown as means ± S.D. of the number of experiments indicated. Statistical evaluation was performed by Student's *t*-test. A *P*-value of < 0.05 was considered statistically significant.

## SUPPLEMENTARY MATERIAL AND FIGURE



## References

[1] Verrill MW, Judson IR, Wiltshaw E, Thomas JM, Harmer CL, Fisher C (1997). The use of paediatric chemotherapy protocols at full dose is both a rational and feasible treatment strategy in adults with Ewing's family tumours. Ann Oncol.

[2] Riggi N, Stamenkovic I (2007). The Biology of Ewing sarcoma. Cancer Lett.

[3] Ludwig JA (2008). Ewing sarcoma: historical perspectives, current state-of-the-art, and opportunities for targeted therapy in the future. Curr Opin Oncol.

[4] Esiashvili N, Goodman M, Marcus RB (2008). Changes in incidence and survival of Ewing sarcoma patients over the past 3 decades: surveillance epidemiology and end results data. J Pediatr Hematol Oncol.

[5] Lissat A, Chao MM, Kontny U (2012). Targeted therapy in Ewing sarcoma. ISRN Oncol.

[6] Hunold A, Weddeling N, Paulussen M, Ranft A, Liebscher C, Jurgens H (2006). Topotecan and cyclophosphamide in patients with refractory or relapsed Ewing tumors. Pediatr Blood Cancer.

[7] Ladenstein R, Potschger U, Le Deley MC, Whelan J, Paulussen M, Oberlin O, van den Berg H, Dirksen U, Hjorth L, Michon J, Lewis I, Craft A, Jurgens H (2010). Primary disseminated multifocal Ewing sarcoma: results of the Euro-EWING 99 trial. J Clin Oncol.

[8] Suva ML, Riggi N, Stehle JC, Baumer K, Tercier S, Joseph JM, Suva D, Clement V, Provero P, Cironi L, Osterheld MC, Guillou L, Stamenkovic I (2009). Identification of cancer stem cells in Ewing's sarcoma. Cancer Res.

[9] Dela Cruz FS (2013). Cancer stem cells in pediatric sarcomas. Front Oncol.

[10] Ross KA, Smyth NA, Murawski CD, Kennedy JG (2013). The biology of ewing sarcoma. ISRN Oncol.

[11] Subbiah V, Anderson P, Lazar AJ, Burdett E, Raymond K, Ludwig JA (2009). Ewing's sarcoma: standard and experimental treatment options. Curr Treat Options Oncol.

[12] Sonnemann J, Dreyer L, Hartwig M, Palani CD, Hong le TT, Klier U, Broker B, Volker U, Beck JF (2007). Histone deacetylase inhibitors induce cell death and enhance the apoptosis-inducing activity of TRAIL in Ewing's sarcoma cells. J Cancer Res Clin Oncol.

[13] Schaefer KL, Eisenacher M, Braun Y, Brachwitz K, Wai DH, Dirksen U, Lanvers-Kaminsky C, Juergens H, Herrero D, Stegmaier S, Koscielniak E, Eggert A, Nathrath M (2008). Microarray analysis of Ewing's sarcoma family of tumours reveals characteristic gene expression signatures associated with metastasis and resistance to chemotherapy. Eur J Cancer.

[14] Mackintosh C, Madoz-Gurpide J, Ordonez JL, Osuna D, Herrero-Martin D (2010). The molecular pathogenesis of Ewing's sarcoma. Cancer Biol Ther.

[15] Nakatani F, Ferracin M, Manara MC, Ventura S, Del Monaco V, Ferrari S, Alberghini M, Grilli A, Knuutila S, Schaefer KL, Mattia G, Negrini M, Picci P (2012). miR-34a predicts survival of Ewing's sarcoma patients and directly influences cell chemo-sensitivity and malignancy. J Pathol.

[16] Iida K, Fukushi J, Matsumoto Y, Oda Y, Takahashi Y, Fujiwara T, Fujiwara-Okada Y, Hatano M, Nabashima A, Kamura S, Iwamoto Y (2013). miR-125b develops chemoresistance in Ewing sarcoma/primitive neuroectodermal tumor. Cancer Cell Int.

[17] Fulda S (2013). How to target apoptosis signaling pathways for the treatment of pediatric cancers. Front Oncol.

[18] Gajate C, Mollinedo F (2002). Biological activities, mechanisms of action and biomedical prospect of the antitumor ether phospholipid ET-18-OCH3 (Edelfosine), a proapoptotic agent in tumor cells. Curr Drug Metab.

[19] Mollinedo F, Gajate C, Martin-Santamaria S, Gago F (2004). ET-18-OCH3 (edelfosine): a selective antitumour lipid targeting apoptosis through intracellular activation of Fas/CD95 death receptor. Curr Med Chem.

[20] van Blitterswijk WJ, Verheij M (2008). Anticancer alkylphospholipids: mechanisms of action, cellular sensitivity and resistance, and clinical prospects. Curr Pharm Des.

[21] Mollinedo F (2014). Editorial: Antitumor alkylphospholipid analogs: a promising and growing family of synthetic cell membrane-targeting molecules for cancer treatment. Anticancer Agents Med Chem.

[22] Leonard R, Hardy J, van Tienhoven G, Houston S, Simmonds P, David M, Mansi J (2001). Randomized, double-blind, placebo-controlled, multicenter trial of 6% miltefosine solution, a topical chemotherapy in cutaneous metastases from breast cancer. J Clin Oncol.

[23] Mollinedo F (2007). Antitumor ether lipids: proapoptotic agents with multiple therapeutic indications. Expert Opin Ther Patents.

[24] Fensterle J, Aicher B, Seipelt I, Teifel M, Engel J (2014). Current view on the mechanism of action of perifosine in cancer. Anticancer Agents Med Chem.

[25] Mollinedo F, Fernandez-Luna JL, Gajate C, Martin-Martin B, Benito A, Martinez-Dalmau R, Modolell M (1997). Selective induction of apoptosis in cancer cells by the ether lipid ET-18-OCH3 (Edelfosine): molecular structure requirements, cellular uptake, and protection by Bcl-2 and Bcl-XL. Cancer Res.

[26] Gajate C, Fonteriz RI, Cabaner C, Alvarez-Noves G, Alvarez-Rodriguez Y, Modolell M, Mollinedo F (2000). Intracellular triggering of Fas, independently of FasL, as a new mechanism of antitumor ether lipid-induced apoptosis. Int J Cancer.

[27] Gajate C, Mollinedo F (2007). Edelfosine and perifosine induce selective apoptosis in multiple myeloma by recruitment of death receptors and downstream signaling molecules into lipid rafts. Blood.

[28] Mollinedo F, de la Iglesia-Vicente J, Gajate C, Estella-Hermoso de Mendoza A, Villa-Pulgarin JA, Campanero MA, Blanco-Prieto MJ (2010). Lipid raft-targeted therapy in multiple myeloma. Oncogene.

[29] Mollinedo F, de la Iglesia-Vicente J, Gajate C, Estella-Hermoso de Mendoza A, Villa-Pulgarin JA, de Frias M, Roue G, Gil J, Colomer D, Campanero MA, Blanco-Prieto MJ (2010). *In vitro* and *in vivo* selective antitumor activity of Edelfosine against mantle cell lymphoma and chronic lymphocytic leukemia involving lipid rafts. Clin Cancer Res.

[30] Gajate C, Del Canto-Janez E, Acuna AU, Amat-Guerri F, Geijo E, Santos-Beneit AM, Veldman RJ, Mollinedo F (2004). Intracellular triggering of Fas aggregation and recruitment of apoptotic molecules into Fas-enriched rafts in selective tumor cell apoptosis. J Exp Med.

[31] Gajate C, Gonzalez-Camacho F, Mollinedo F (2009). Involvement of raft aggregates enriched in Fas/CD95 death-inducing signaling complex in the antileukemic action of edelfosine in Jurkat cells. PLoS ONE.

[32] Gajate C, Mollinedo F (2001). The antitumor ether lipid ET-18-OCH3 induces apoptosis through translocation and capping of Fas/CD95 into membrane rafts in human leukemic cells. Blood.

[33] Mollinedo F, Gajate C (2010). Lipid rafts and clusters of apoptotic signaling molecule-enriched rafts in cancer therapy. Future Oncol.

[34] Gajate C, Mollinedo F (2014). Lipid rafts, endoplasmic reticulum and mitochondria in the antitumor action of the alkylphospholipid analog edelfosine. Anticancer Agents Med Chem.

[35] Gajate C, Matos-da-Silva M, Dakir EL, Fonteriz RI, Alvarez J, Mollinedo F (2012). Antitumor alkyl-lysophospholipid analog edelfosine induces apoptosis in pancreatic cancer by targeting endoplasmic reticulum. Oncogene.

[36] Nieto-Miguel T, Fonteriz RI, Vay L, Gajate C, Lopez-Hernandez S, Mollinedo F (2007). Endoplasmic reticulum stress in the proapoptotic action of edelfosine in solid tumor cells. Cancer Res.

[37] Estella-Hermoso de Mendoza A, Campanero MA, de la Iglesia-Vicente J, Gajate C, Mollinedo F, Blanco-Prieto MJ (2009). Antitumor alkyl ether lipid edelfosine: tissue distribution and pharmacokinetic behavior in healthy and tumor-bearing immunosuppressed mice. Clin Cancer Res.

[38] Gajate C, Santos-Beneit A, Modolell M, Mollinedo F (1998). Involvement of c-Jun NH2-terminal kinase activation and c-Jun in the induction of apoptosis by the ether phospholipid 1-*O*-octadecyl-2-*O*-methyl-*rac*-glycero-3-phosphocholine. Mol Pharmacol.

[39] Harris SA, Enger RJ, Riggs BL, Spelsberg TC (1995). Development and characterization of a conditionally immortalized human fetal osteoblastic cell line. J Bone Miner Res.

[40] Subramaniam M, Jalal SM, Rickard DJ, Harris SA, Bolander ME, Spelsberg TC (2002). Further characterization of human fetal osteoblastic hFOB 1. 19 and hFOB/ER alpha cells: bone formation *in vivo* and karyotype analysis using multicolor fluorescent *in situ* hybridization. J Cell Biochem.

[41] Cuesta-Marban A, Botet J, Czyz O, Cacharro LM, Gajate C, Hornillos V, Delgado J, Zhang H, Amat-Guerri F, Acuna AU, McMaster CR, Revuelta JL, Zaremberg V (2013). Drug uptake, lipid rafts, and vesicle trafficking modulate resistance to an anticancer lysophosphatidylcholine analogue in yeast. J Biol Chem.

[42] Mollinedo F, Fernandez M, Hornillos V, Delgado J, Amat-Guerri F, Acuna AU, Nieto-Miguel T, Villa-Pulgarin JA, Gonzalez-Garcia C, Cena V, Gajate C (2011). Involvement of lipid rafts in the localization and dysfunction effect of the antitumor ether phospholipid edelfosine in mitochondria. Cell Death Dis.

[43] Klee M, Pimentel-Muinos FX (2005). Bcl-XL specifically activates Bak to induce swelling and restructuring of the endoplasmic reticulum. J Cell Biol.

[44] Breckenridge DG, Stojanovic M, Marcellus RC, Shore GC (2003). Caspase cleavage product of BAP31 induces mitochondrial fission through endoplasmic reticulum calcium signals, enhancing cytochrome c release to the cytosol. J Cell Biol.

[45] Li J, Lee AS (2006). Stress induction of GRP78/BiP and its role in cancer. Curr Mol Med.

[46] Gee KR, Brown KA, Chen WN, Bishop-Stewart J, Gray D, Johnson I (2000). Chemical and physiological characterization of fluo-4 Ca^2+^-indicator dyes. Cell Calcium.

[47] Iwasawa R, Mahul-Mellier AL, Datler C, Pazarentzos E, Grimm S (2011). Fis1 and Bap31 bridge the mitochondria-ER interface to establish a platform for apoptosis induction. EMBO J.

[48] Nishitoh H, Matsuzawa A, Tobiume K, Saegusa K, Takeda K, Inoue K, Hori S, Kakizuka A, Ichijo H (2002). ASK1 is essential for endoplasmic reticulum stress-induced neuronal cell death triggered by expanded polyglutamine repeats. Genes Dev.

[49] Grier HE, Krailo MD, Tarbell NJ, Link MP, Fryer CJ, Pritchard DJ, Gebhardt MC, Dickman PS, Perlman EJ, Meyers PA, Donaldson SS, Moore S, Rausen AR (2003). Addition of ifosfamide and etoposide to standard chemotherapy for Ewing's sarcoma and primitive neuroectodermal tumor of bone. N Engl J Med.

[50] Whelan J, Khan A, Sharma A, Rothermundt C, Dileo P, Michelagnoli M, Seddon B, Strausss S (2012). Interval compressed vincristine, doxorubicin, cyclophosphamide alternating with ifosfamide, etoposide in patients with advanced Ewing's and other Small Round Cell Sarcomas. Clin Sarcoma Res.

[51] Mollinedo F, Gajate C (2003). Microtubules, microtubule-interfering agents and apoptosis. Apoptosis.

[52] Mollinedo F (2005). Survival and apoptotic signals in the action of microtubule-targeting antitumor drugs. IDrugs.

[53] Tacar O, Sriamornsak P, Dass CR (2013). Doxorubicin: an update on anticancer molecular action, toxicity and novel drug delivery systems. J Pharm Pharmacol.

[54] Emadi A, Jones RJ, Brodsky RA (2009). Cyclophosphamide and cancer: golden anniversary. Nat Rev Clin Oncol.

[55] He H, Ke R, Lin H, Ying Y, Liu D, Luo Z (2015). Metformin, an old drug, brings a new era to cancer therapy. Cancer J.

[56] Yamamuro A, Kishino T, Ohshima Y, Yoshioka Y, Kimura T, Kasai A, Maeda S (2011). Caspase-4 directly activates caspase-9 in endoplasmic reticulum stress-induced apoptosis in SH-SY5Y cells. J Pharmacol Sci.

[57] Ledgerwood EC, Morison IM (2009). Targeting the apoptosome for cancer therapy. Clin Cancer Res.

[58] Yao C, Wei JJ, Wang ZY, Ding HM, Li D, Yan SC, Yang YJ, Gu ZP (2013). Perifosine induces cell apoptosis in human osteosarcoma cells: new implication for osteosarcoma therapy?. Cell Biochem Biophys.

[59] Bailey HH, Mahoney MR, Ettinger DS, Maples WJ, Fracasso PM, Traynor AM, Erlichman C, Okuno SH (2006). Phase II study of daily oral perifosine in patients with advanced soft tissue sarcoma. Cancer.

[60] Knowling M, Blackstein M, Tozer R, Bramwell V, Dancey J, Dore N, Matthews S, Eisenhauer E (2006). A phase II study of perifosine (D-21226) in patients with previously untreated metastatic or locally advanced soft tissue sarcoma: A National Cancer Institute of Canada Clinical Trials Group trial. Invest New Drugs.

[61] Mollinedo F, Gajate C, Morales AI, del Canto-Janez E, Justies N, Collia F, Rivas JV, Modolell M, Iglesias A (2009). Novel anti-inflammatory action of edelfosine lacking toxicity with protective effect in experimental colitis. J Pharmacol Exp Ther.

[62] Del Barco S, Vazquez-Martin A, Cufi S, Oliveras-Ferraros C, Bosch-Barrera J, Joven J, Martin-Castillo B, Menendez JA (2011). Metformin: multi-faceted protection against cancer. Oncotarget.

[63] Pierotti MA, Berrino F, Gariboldi M, Melani C, Mogavero A, Negri T, Pasanisi P, Pilotti S (2013). Targeting metabolism for cancer treatment and prevention: metformin, an old drug with multi-faceted effects. Oncogene.

[64] Gritti M, Wurth R, Angelini M, Barbieri F, Peretti M, Pizzi E, Pattarozzi A, Carra E, Sirito R, Daga A, Curmi PM, Mazzanti M, Florio T (2014). Metformin repositioning as antitumoral agent: selective antiproliferative effects in human glioblastoma stem cells, via inhibition of CLIC1-mediated ion current. Oncotarget.

[65] Quattrini I, Conti A, Pazzaglia L, Novello C, Ferrari S, Picci P, Benassi MS (2014). Metformin inhibits growth and sensitizes osteosarcoma cell lines to cisplatin through cell cycle modulation. Oncol Rep.

[66] Cufi S, Corominas-Faja B, Vazquez-Martin A, Oliveras-Ferraros C, Dorca J, Bosch-Barrera J, Martin-Castillo B, Menendez JA (2012). Metformin-induced preferential killing of breast cancer initiating CD44+CD24-/low cells is sufficient to overcome primary resistance to trastuzumab in HER2+ human breast cancer xenografts. Oncotarget.

[67] Morgillo F, Sasso FC, Della Corte CM, Vitagliano D, D'Aiuto E, Troiani T, Martinelli E, De Vita F, Orditura M, De Palma R, Ciardiello F (2013). Synergistic effects of metformin treatment in combination with gefitinib, a selective EGFR tyrosine kinase inhibitor, in LKB1 wild-type NSCLC cell lines. Clin Cancer Res.

[68] Lin YC, Wu MH, Wei TT, Huang WC, Huang LY, Lin YT, Chen CC (2014). Metformin sensitizes anticancer effect of dasatinib in head and neck squamous cell carcinoma cells through AMPK-dependent ER stress. Oncotarget.

[69] Vujic I, Sanlorenzo M, Posch C, Esteve-Puig R, Yen AJ, Kwong A, Tsumura A, Murphy R, Rappersberger K, Ortiz-Urda S (2015). Metformin and trametinib have synergistic effects on cell viability and tumor growth in NRAS mutant cancer. Oncotarget.

[70] Zi FM, He JS, Li Y, Wu C, Yang L, Yang Y, Wang LJ, He DH, Zhao Y, Wu WJ, Zheng GF, Han XY, Huang H (2015). Metformin displays anti-myeloma activity and synergistic effect with dexamethasone in *in vitro* and *in vivo* xenograft models. Cancer Lett.

[71] Mollinedo F, Martinez-Dalmau R, Modolell M (1993). Early and selective induction of apoptosis in human leukemic cells by the alkyl-lysophospholipid ET-18-OCH3. Biochem Biophys Res Commun.

[72] Gajate C, Barasoain I, Andreu JM, Mollinedo F (2000). Induction of apoptosis in leukemic cells by the reversible microtubule-disrupting agent 2-methoxy-5-(2′,3′,4′-trimethoxyphenyl)-2,4,6-cycloheptatrien-1-one: protection by Bcl-2 and Bcl-XL and cell cycle arrest. Cancer Res.

[73] Gajate C, An F, Mollinedo F (2003). Rapid and selective apoptosis in human leukemic cells induced by Aplidine through a Fas/CD95- and mitochondrial-mediated mechanism. Clin Cancer Res.

[74] Gajate C, Santos-Beneit AM, Macho A, Lazaro M, Hernandez-De Rojas A, Modolell M, Munoz E, Mollinedo F (2000). Involvement of mitochondria and caspase-3 in ET-18-OCH3-induced apoptosis of human leukemic cells. Int J Cancer.

