# Supplementary material for: Endoplasmic reticulum targeting in Ewing's sarcoma by the alkylphospholipid analog edelfosine
Source: Oncotarget. 2015 May 9;6(16):14596–613. doi: 10.18632/oncotarget.4053 (PMC4546490; doi:10.18632/oncotarget.4053)
Supplement: Supplementary file 1 [file oncotarget-06-14596-s001.pdf]

Endoplasmic reticulum targeting in Ewing's sarcoma by the alkylphospholipid analog edelfosine

Supplementary Material

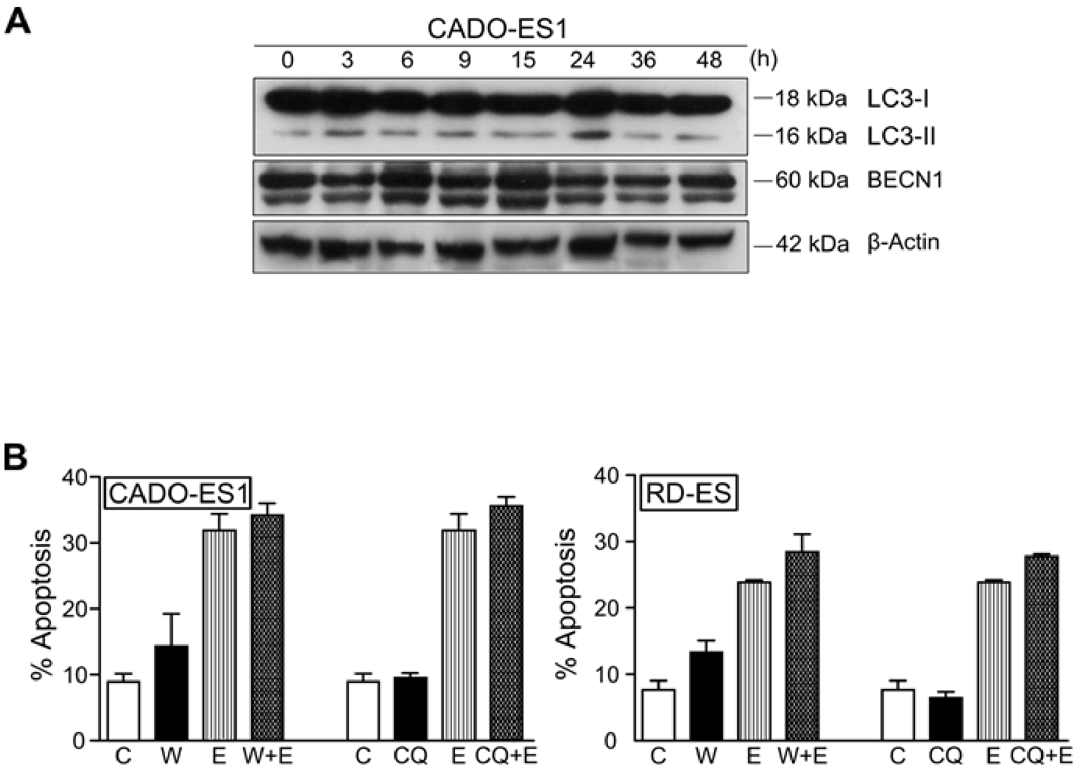

Supplementary Figure S1. Minor autophagic response induced by edelfosine in ES cells. *A*, CADO-ES1 cells were incubated in the absence (*0 h*) or presence of 10 μM edelfosine at the shown times, and then cells were analyzed by immunoblotting with antibodies directed against the indicated proteins. β-Actin was used as a loading control. Data are representative of three experiments performed. *B*, ES cells were incubated in the absence (*C*) or the presence of 500 nM wortmannin (W), 30 μM chloroquine (CQ), 10 μM edelfosine (E), 500 nM wortmannin + 10 μM edelfosine (W+E) or 30 μM chloroquine + 10 μM edelfosine (CQ+E) for 24 h, and then apoptosis was determined as the percentage of cells in the sub-G_1 region (hypodiploidy) analyzed by flow cytometry. Data are means ± SD of three independent experiments.
